# Multifunctional Stretchable Conductive Woven Fabric Containing Metal Wire with Durable Structural Stability and Electromagnetic Shielding in the X-Band

**DOI:** 10.3390/polym12020399

**Published:** 2020-02-10

**Authors:** Yong Wang, Stuart Gordon, Thomas Baum, Zhenzhen Xu

**Affiliations:** 1 College of Textiles and Garments, Anhui Polytechnic University, Wuhu 241000, China; 2 CSIRO Manufacturing, Waurn Ponds, VIC 3216, Australia; stuart.gordon@csiro.au; 3 Defence Science and Technology Group, Fishermans Bend, Melbourne, VIC 3207, Australia; thomas.baum@rmit.edu.au; 4 School of Electrical and Computer Engineering, RMIT University, Melbourne, VIC 3000, Australia

**Keywords:** electromagnetic shielding fabric, elastic-conductive composite yarn, stretchable fabric, multifunctional textiles, protective device, cyclic elastic recovery behavior

## Abstract

Elastomeric, conductive composite yarns have recently received attention around the opportunity for them to offer special protective fields. A straightforward approach for fabricating tri-component elastic-conductive composite yarns (t-ECCYs) containing stainless steel wire (SSW) was proposed previously. This work mainly focuses on the electromagnetic shielding effectiveness (EMSE) of weft-stretchable woven fabric containing t-ECCY over the X-band under different testing conditions, e.g., single step-by-step elongation, cyclic stretch and lamination events. Results showed that a woven cotton fabric with weft yarn of t-ECCY not only exhibited superior weft stretch-ability to a higher elongation (>40%) compared with a pure cotton control but also had an acceptable 15-cyclic stability with 80% shape recovery retention. The t-ECCY weft fabric was effective in shielding electromagnetic radiation, and its EMSE was also enhanced at elevated elongations during stretch at parallel polarization of EM waves. There was also no decay in EMSE before and after the t-ECCY fabric was subject to 15 stretch cycles at extension of 20%. In addition, a 90° by 90° cross lamination of t-ECCY fabric remarkably improved the EMSE compared to a 0°/90° one. The scalable fabrication strategy and excellent EMSE seen in t-ECCY-incorporated fabrics represent a significant step forward in protective fields.

## 1. Introduction

Electromagnetic interference (EMI) is a new but ubiquitous source of radiation pollution, which not only affects the normal function of sensitive electronic equipment and systems but also increasingly has become an environmental and health concern mainly due to a rapid increase in use of telecommunication, digital devices, coil components and electromagnets [1,2,3,4]. There is therefore an important realistic significance and practical value in finding solutions to these problems and shielding both people and their devices (and information) from these emissions.

The concept of smart textiles has gained considerable momentum over the last decade [5,6,7,8,9,10,11], and the implementation of such textiles in the form of fibers, yarns or fabrics containing conductive elements is crucial in a wide range of applications such as telecommunications, medical monitoring and therapy, electromagnetic shielding, electro-thermal device, and aesthetic design. Recently, the development of conductive fabrics that provide environmental and health care protection from EM waves has entered mainstream consciousness [12]. They can be extensively used in radar and thermal camouflage in military, civilian, and other fields. Generally, the main EM-shielding fabrics can be prepared using conductive yarns and/or textiles with enhanced conductivity obtained via surface treatments, e.g., conductive coating, ionic/electroless plating, vacuum metallization [13]. For EM shielding yarns, metal wires incorporated into the yarn are often considered to be the best and most straightforward EM shielding material because of their excellent conductivity and the fabric′s permeability [14]. Compared to traditional stiff metallic materials, yarns and/or fabrics containing metallic staple fibers/continuous wires have gained considerable interest recently [14,15,16,17,18]. Yarns with a range of different conductive fibers and wires have been examined including stainless steel [5,7,19,20,21,22,23], copper [23,24], silver-coated wire [25]. Fabrics containing these components offer a great opportunity to develop a new wave of EM shielding textiles due to their versatility, flexibility and 3D conformability to any desired apparel. The use of yarns or fabrics with surface treatments, e.g., conductive paints [26,27], vacuum metallization, ionic/electroless plating, and cathode sputtering, whilst also flexible in terms of these desired properties and affording shielding of EM radiation, may not be strong enough to withstand the rigors that occur during wear.

Based on current literature on the EMI shielding of woven/knitted fabrics containing continuous metal wires and/or metallic fibers, it is noted that factors such as the type of metal, its thickness, metallic content and its distribution and orientation of within a yarn and/or fabric, along with fabric factors such as weave construction, warp/weft density, the lamination layers and lamination angles have considerable effects on the EMSE values of the resultant composite fabrics [13,14,15,16,17,18,19,20,21,22,23,24,25,26,27]. However, work reported so far has mostly measured EM radiation in the low frequency range (usually 0–3 GHz) and fabrics without any elastic features. The need to shift bandwidths to the higher frequency levels as a result of the further advancement/requirement in technologies is inevitable. In recent years, the X-band frequency region has been applied to a wide range of applications in satellite communication, radar navigation, air traffic control, detection and location of objects, stealth technology in military combat, marine functions, environmental monitoring, geological survey and many other fields. With the advent of these applications, workers are now exposed to these frequencies in their workplace. These frequencies also have some adverse effects on human health. Thus, investigations on the EMSE performance of textile structures with excellent elastic properties over X-band have practical and meaningful application.

So far, there have been few advances in the development of elastic EM shielding fabric structures in higher (e.g., X-band) frequency range. For example, Lin et al. successfully prepared electromagnetic shielding/far-infrared elastic warp-knitted fabrics by using PET filament and rubber threads as warps and charcoal/stainless steel wrap yarns as wefts. The fabrics displayed reasonable electromagnetic shielding efficiency at lower frequency range up to 3 GHz and super elastic response rates following five cycles of stretch with 50% preset elongation [12]. The EMSE values of these fabrics following test cycles and single continuous elongations were not taken into account. Gupta et al. measured the EMI shielding of ultra-lightweight woven fabrics containing staple stainless-steel fiber/polyester hybrid yarns over the X- and Ku-bands [28]. However, these fabrics were also non-elastic. Pandey et al. made a comprehensive evaluation of the EMSE behavior of three-dimensional woven fabrics with copper-based hybrid yarn in the X-band frequency range [29]. Similarly, these fabrics were also inherently inelastic. Thus, one purpose of this work is to provide information around the above-mentioned gap in the analysis of conductive fabrics with elastic properties.

To simultaneously integrate these intriguing properties for a woven stretchable fabric, including elasticity, flexibility, cyclic durability, excellent EMI shielding performance, and easy processing, herein, we prepared a woven fabric incorporating tri-component, elastic-conductive composite yarns (t-ECCYs) [30,31,32,33,34] along its weft direction, which can be applied for radar and thermal camouflage in military applications. The EMSE values of the fabric in the X-band frequency range under different testing conditions, e.g., single elongation, cyclic stretching, and lamination, were systematically investigated. In particular, the effect of the anisotropy of fabric structure, namely, the orientation of t-ECCY within the fabric, was clarified. In addition, a pure cotton woven fabric (control sample) was fabricated as a control. This work puts forward a promising and scalable strategy for large-scale, low-cost fabrication of multifunctional stretchable and wearable protective devices.

## 2. Experimental Procedures

### 2.1. Materials and Sample Preparation

**Figure 1:**
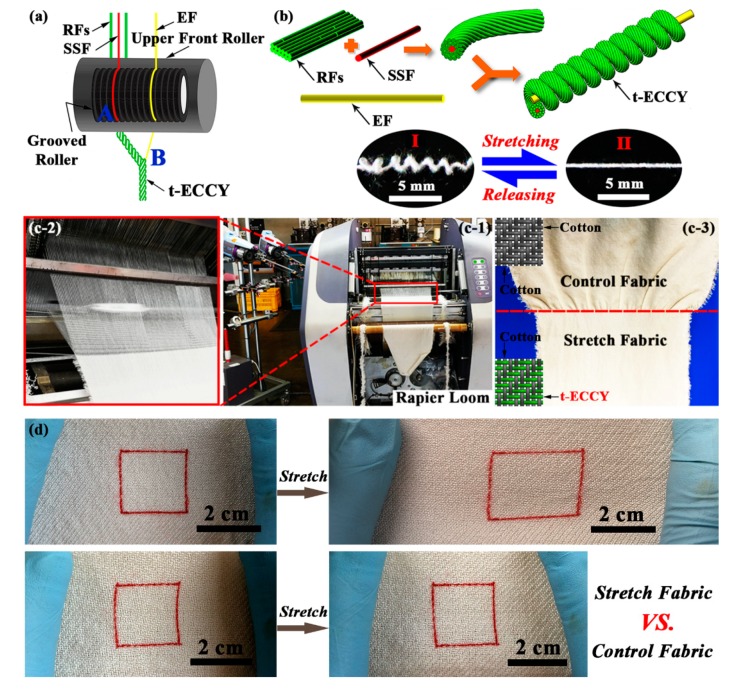
(**a**,**b**) Forming mechanism of tri-component elastic-conductive composite yarns (t-ECCYs) on a modified ring spun frame; (**c**) a rapier loom for producing woven fabrics and the corresponding woven samples; (**d**) photographs of the stretchable woven fabric and control sample at initial and stretched states, respectively.

The t-ECCY used in this study was prepared as per our reported work [30,31,32,33], which employed an elastane filament (EF) of 140 D in the core and a stainless steel filament (SSF) of 30 μm in diameter, combined with the rayon staple fibers (RFs) that were spun around the elastane core. The EF, SSF and RFs were purchased from Haining Kaiwei Textile Co., Ltd., Laiwu Longzhi Metal Yarn Co., Ltd., China, and Xianyang China Resources Textile Co., Ltd., respectively. The resultant yarn has a more bulking surface profile compared with non-elastane variants. The EF facilitates extension values up to approximately a strain of 300% without distortion (Figure 1a,b).

To explore the potential uses of these yarns, a specified woven fabric was made in an automatic rapier loom (CCI Technologies Inc., Zhejiang, China); see Figure 1c. A 2/2 twill weave was made with a count of 44 ends/inch × 44 picks/inch. The warp and t-ECCY weft yarns had a count of 55 and 43.5 tex, respectively. A woven fabric made from 100% cotton yarns (55 tex in both warp and weft) was prepared as a control. Figure 1d illustrates the superior elasticity of the t-ECCY-incorporated fabric in weft direction compared with the control sample. It is noteworthy to mention that no finishing processes, e.g., sizing/singeing, were applied to these fabric samples before testing.

### 2.2. EMSE Measurement of Woven Fabrics

**Figure 2:**
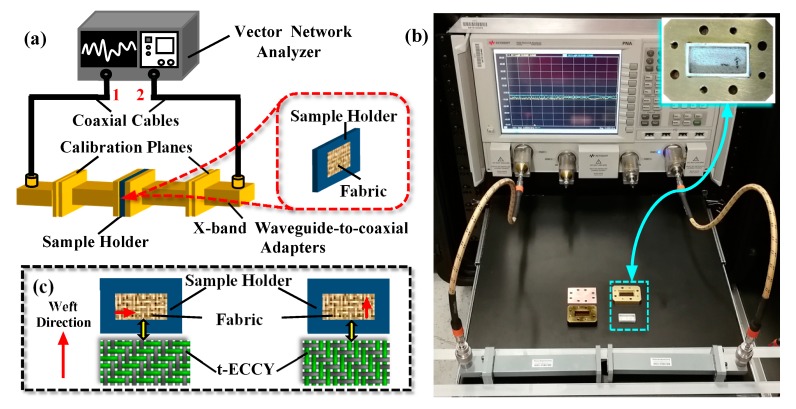
Schematic diagram (**a**) and the real device (**b**) of electromagnetic interference (EMI) shielding measurement system in the X-band frequency range; (**c**) preparation of fabric samples along two directions.

The EMSE performance of woven fabrics was investigated in X-band (8.2–12.4 GHz) frequency range under various testing conditions; the fabric in an un-tensioned state without load, in a single stretch at different elongations, after a cyclic stretch routine and laminated in different directions. Details of these states as applied to the fabric are described as follows: The EMSE of testing specimens of the stretchable fabric were measured in an ‘initial’ state without load, at different monotonous elongations (0%, 10%, 40%), following 15 cyclic tensile tests at 20% extension with a preload of 1 N and in two-layer laminations of different lamination angles (0°/0°, 0°/90° and 90°/90°). Measurements were made over the X-band range using a Keysight-N5225A network analyzer (Keysight Technologies Inc., Santa Rosa, CA, USA). Fabric specimens were stuck into a rectangular waveguide (PEWCA1032), sample holder (Figure 2a). All specimens had the dimension of 2.28 cm (0.9 inch) × 1.14 cm (0.45 inch). A photograph of the testing setup is displayed in Figure 2b. Measurements were performed along two directions, i.e., perpendicular and parallel, as illustrated in Figure 2c.

Prior to the test measurements, a Thru-Reflect-Line (TRL) calibration was performed in order to remove the errors [35]. A total 673 data points were taken for each sample, and the corresponding *S*-parameters (*S*_11_, *S*_12_, *S*_22_ and *S*_21_) were recorded over this frequency range. For each formulation, at least three specimens were fabricated and tested. It is worth mentioning that the EMSE of the control fabric was also studied in X-band frequency range for comparison.

### 2.3. Elastic Recovery Response of Weft-Stretchable Woven Fabric

**Figure 3:**
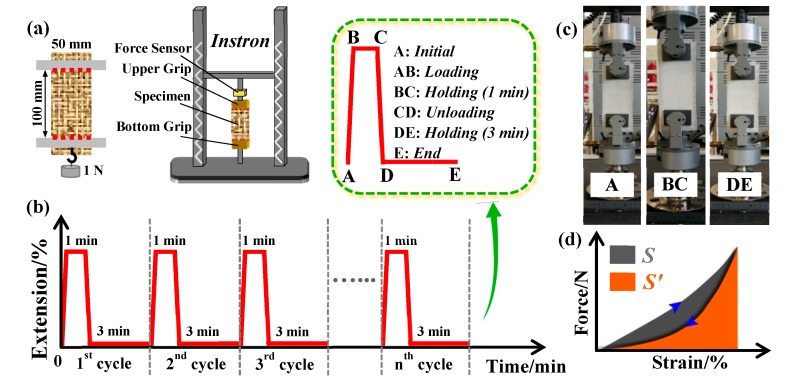
(**a**) The preload-imposing mode and placement of fabric samples on an Instron tensile testing device; (**b**,**c**) schematic and real photographs of fabric movement during tensile loading cycles, respectively; (**d**) dynamic work recovery (*DWR*) curves of a fabric.

Based on the demand of wear and actual use, the as-prepared weft stretchable woven fabric should possess a better long-term structural stability following repeated stretch and recovery. Considering the measured stretch elasticity of fabric (>40%) and the expansion across different body parts during the human movement [36,37], i.e., cyclic movements between 10% and 50%, the elastic recovery of fabric at 20% extension was examined following 1, 10 and 15 cyclic tensile tests. The cyclic test procedure was undertaken as per the China standard FZ/T 01034-2008 [38]: The fabric (50 mm width × 100 mm length) with a pretension of 1 N was tested with an Instron 5567 tensile device (made in USA) to stretch the sample to 20% extension at a speed of 100 mm/min, held at this extension for 1 min; then, the tension was unloaded at the same speed to initial position for 3 min relaxation; after that, the fabric was reloaded with the same preload to measure its elongation length. The dynamic cyclic tests were performed using the same processes (Figure 3a,b). Each fabric specimen was tested 3 times. Photos of the fabric captured during the test cycles are shown in Figure 3c. It is noteworthy that the preload-imposing mode and the placement of fabric on this tensile device are similar to our previous report concerning about yarns by Wang et al. [32]. Two indexes, that is, elastic recovery ratio (*ERR*) and plastic deformation ratio (*PDR*), can be calculated:

(1) $$ERR\;(\%)=\frac{L_{01}-{L_{0}}^{\prime }}{L_{01}-L_{0}}\times 100,\;PDR\;(\%)=\frac{{L_{0}}^{\prime }-L_{0}}{L_{0}}\times 100$$

where *L*_0_ is fabric length when loaded pretension (mm); *L*_0_′ is fabric length after tests when loaded pretension (mm); and *L*_01_ is initial length × (1 + extension/%) (mm).

Apart from the above static elastic recovery aimed to analyze a fabric’s dimensional stability, the dynamic work recovery (*DWR*) provides a model for garment′s response to human body movements [37]. Most textile fabrics will produce a hysteresis loop during the tensile loading-unloading tests [39,40]. The higher the hysteresis area, the higher the energy loss. The value of *DWR* can be calculated from a fabric’s stretch-recovery tensile curve (from Figure 3d):

(2) $$DWR(\%)=\frac{\mathrm{area}\;\mathrm{under}\;\mathrm{the}\;\mathrm{unloading}\;\mathrm{curve}}{\mathrm{area}\;\mathrm{under}\;\mathrm{the}\;\mathrm{loading}\;\mathrm{curve}}\times 100=\frac{S^{\prime }}{S}\times 100$$

## 3. EMI Shielding Mechanism and Data Analysis

**Figure 4:**
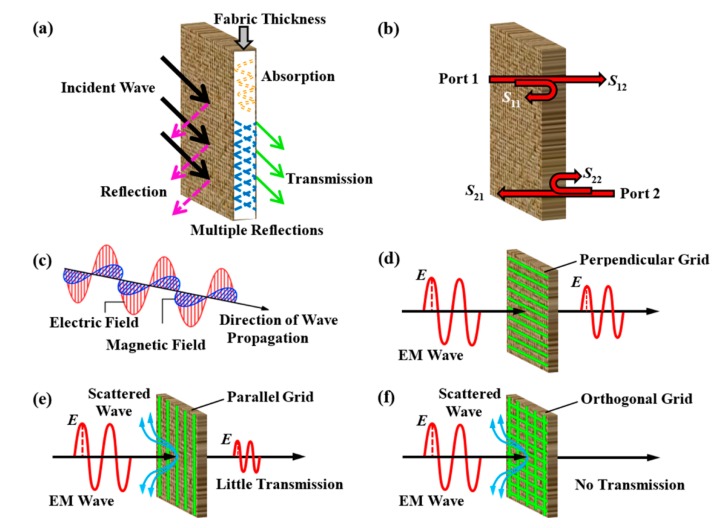
(**a**) Graphical representation of an EM wave on passing through an EM shielding fabric; (**b**) Schematic diagram of *S*-parameters; (**c**) EM wave with its electric and magnetic components; (**d**–**f**) The theoretical models showing EM wave incidences when the *E* field perpendicular to conductive yarn (perpendicular polarization), when the *E* field parallel to conductive yarn (parallel polarization) and when the conductive elements are in both directions, respectively.

EMSE is defined as the attenuation of propagating EM waves produced by a shielding material [28]. A schematic of an EM wave passing through a shielding fabric is graphically shown in Figure 4a. According to Shelkunoff′s theory, total shielding effectiveness (*SE*_T_) can be described as the sum of contribution due to reflection (*SE*_R_), absorption (*SE*_A_) and multiple internal reflections (*SE*_M_) [41,42]:

(3) $$SE_{T}(\mathrm{dB})=SE_{R}+SE_{A}+SE_{M}$$

The *SE*_R_ is often viewed as a main mechanism which requires the presence of mobile charge carriers like electrons or holes; *SE*_A_ is realized by electric or magnetic dipoles that interact with EM fields in the radiation [43]. The *SE*_M_ is a correction term whose value may be positive, negative or zero, and it can be expressed in terms of *SE*_A_ [41]:

(4) $$SE_{M}=20log\left(1-10^{-\left(\frac{SE_{A}}{10}\right)}\right)$$

Therefore, when *SE*_A_ > 10 dB, the loss due to *SE*_M_ is negligible (*SE*_M_ ≈ 0).

The *S*-parameters of two-port vector network analyzer represent reflectance and transmittance, respectively, which includes the intensities of incident wave *S*_11_ (or *S*_22_) and transmitted wave *S*_12_ (or *S*_21_). As discussed above, EMSE has three distributions: reflectivity (*R*), absorptivity (*A*) and the multi-reflecting correction of waves inside the shielding barrier. For a very approximate analysis, the multi-reflecting part could be neglected, particularly for the case when *SE*_A_ > 10 dB. Thus, we could evaluate each contribution for total EMI shielding by the following formulas [44,45]. The values of *I*, *R*, *T* and *A* are the incident, reflected, transmitted and absorbed powers respectively normalized against the incident power.

(5) $$I=1,\;R={\left|S_{11}\right|}^{2}={\left|S_{22}\right|}^{2},\;T={\left|S_{12}\right|}^{2}={\left|S_{21}\right|}^{2},\;A=I-R-T$$

(6) $$SE_{R}=10\log\left(\frac{I}{I-R}\right)=10\log\left(\frac{1}{1-R}\right)=-10\log(1-R)$$

(7) $$SE_{A}=10\log\left(\frac{I-R}{T}\right)=10\log\left(\frac{1-R}{T}\right)=-10\log\left(\frac{T}{1-R}\right)$$

(8) $$SE_{T}=SE_{R}+SE_{A}=10\log\left(\frac{I}{T}\right)=10\log\left(\frac{1}{T}\right)$$

In addition, The *SE*_T_ in dB can be transformed into percentage [46]:

(9) $$SE(\%)=\left(1-\frac{1}{10^{\left(\frac{SE}{10}\right)}}\right)\times 100$$

In general, the metal fiber content and its distribution within the fabric and fabric geometry have a significant effect of the fabric shielding effectiveness. In this paper, only one kind of stretchable woven fabric was fabricated; the warp/weft density and weave texture remained unchanged. Fabrics normally do not possess isotropic EM shielding behavior and typically reflect the yarn orientation during the EMSE measurement [16,47,48]. Here, we focused on the conductive yarn′s distribution to understand the influence mechanism of vertical and horizontal polarization of wave on EMSE.

As illustrated in Figure 4c, an EM wave consists of electric and magnetic components. When EM wave incidences on conducting elements with *E* field being perpendicular to conducting element (Figure 4d), the cumulative losses are due to Lambert cosine rule, the finite ohm current loss in the conducting element and loss due to the inductive capacitive effect. When an EM wave is incident to the conducting element with *E* field of wave parallel to conducting element (Figure 4e), an induced electrical field and a displacement current is generated in the conductor; this causes free electrons in metal to move under an acceleration that emits radiations in all directions, and the conducting element behaves as if it was a new source of EM wave as per the Huygens′s principle. In this situation, a maximum of EM scattering and reflection occurs. When an EM wave falls on a screen having conducting elements in warp and weft directions, transmission is entirely blocked resulting in total EM shielding (Figure 4f) [28].

## 4. Results and Discussion

### 4.1. Elastic Recovery Behavior of Stretchable Fabric Following Cyclic Stretch

#### 4.1.1. Static Elastic Recovery Values of Fabric Following Cyclic Stretch

Investigating of dynamic elastic behavior is an objective evaluation of the stretch and recovery performance of elastic fabrics. Table 1 summarizes the *ERR* and *PDR* values of stretchable fabric after different times of repeating tensile tests. It is demonstrated that the *ERR* decreases when the fabric is subject to cyclic tests, from 94.17% with 1 cycle to 83.33% and 80.83% when the cyclic number goes up to 10 and 15 times respectively, which indicates an acceptable cycling stability with shape recovery retention (>80%) following a higher cycle. Furthermore, the *PDR* values increase with an increase of cyclic times. The fabric suffers from a loss of partial elasticity and a gradual accumulation of plasticity following cyclic tests, whereas a limited effect of elastic recovery happens when it was stretched at a lower cycle.

#### 4.1.2. Dynamic Work Recovery Values of Fabric Following Cyclic Stretch

**Figure 5:**
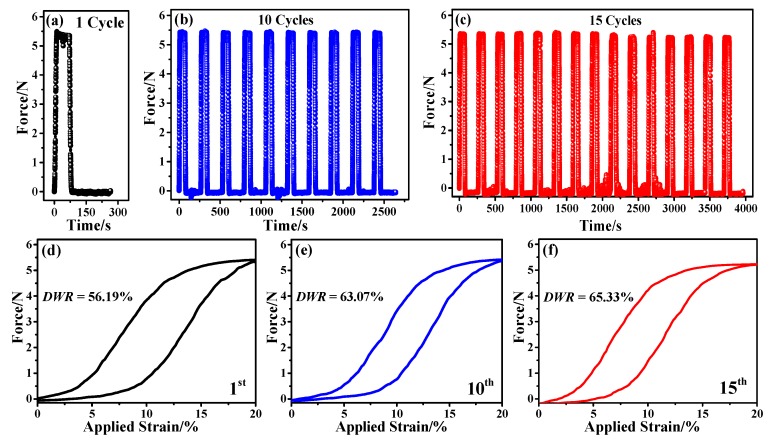
(**a**–**c**) Typical force-time curves of weft-stretchable fabric at an extension of 20% following 1, 10 and 15 cycles, respectively; (**d**–**f**) typical curves with smooth processing and the corresponding *DWR* values at the 1st, 10th and 15th cycle, respectively.

Figure 5a–c shows the typical 1, 10 and 15 cyclic force-time curves at 20% pre-strain of the weft stretchable fabric. As can be seen, the shapes of all test curves following different cycles are almost the same, and the maximum force achieved at consecutive cycles is maintained very well. Specifically, the representative curves at 1st, 10th and 15th cycles are given in Figure 5d–f, respectively. All the shapes of these curves are essentially nonlinear, with an initial concavity and a consequent convexity feature. The plots show the hysteretic behavior of the stretchable fabric, with the force hysteresis at the 1st cycle larger than at the 10th and 15th cycles. When cyclic number increases from 1 to 10 times, the *DWR* increases remarkably from 56.19% to 63.07%. With a further increase to 15 times, the *DWR* increases marginally to 65.33%. When stretched at the 1st cycle, the interlaced structure deformation has been changed, and the status is irreversible causing permanent deformation. Thus, the hysteresis of force in the 1st cycle is distinctive. The hysteresis of force in the 10th and 15th cycle lessens because the above permanent deformations of such fabric are relatively firm with a lower strain of 20% [47]. Besides, the *DWR* values are relatively smaller, which agrees well with the reports by Senthilkumar et al. [37].

### 4.2. EMSE Evaluation of Stretch Fabric in X-band 8.2–12.4 GHz

#### 4.2.1. EMSE of Fabric in Initial State (with Free Load)

In order to clarify the effect of the fabric weaving structure considering its anisotropy, EMSE values of the control and stretchable woven fabrics in initial state at both perpendicular and parallel polarizations are investigated.

**Figure 6:**
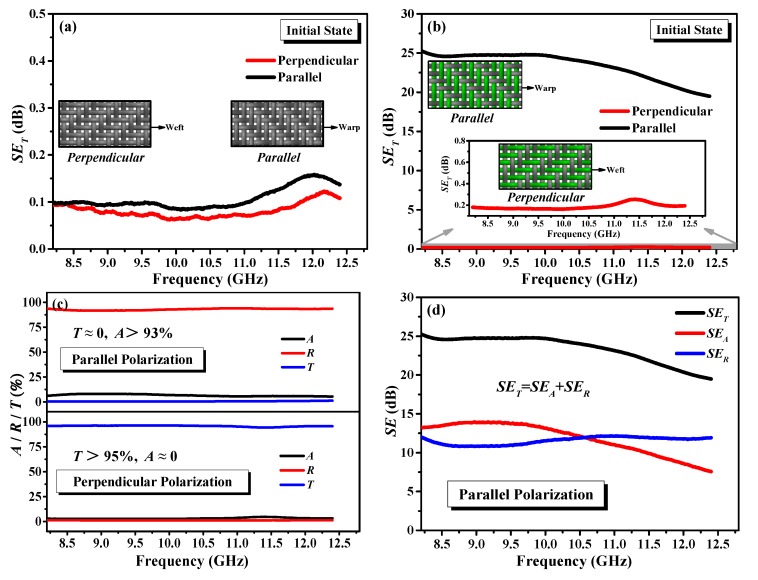
(**a**) Total EMSE results of the (a) control and (**b**) stretchable woven fabrics in respective initial states at perpendicular and parallel polarizations of EM wave over X-band; (**c**) Absorption, reflection and transmission coefficients at perpendicular and parallel polarizations, respectively; (**d**) Absorption and reflection contributions of stretchable fabric at parallel polarization.

The control woven fabric produced from 100% cotton yarns was checked for EMSE at both perpendicular and parallel polarizations of incident EM wave. Its *SE*_T_ response for the two different directions is shown in Figure 6a. The control fabric without SSF exhibits almost no EMI shielding ability for either of the polarizations because cotton is an inherent electrical insulator and transparent to EM radiation. Results in Figure 6b suggest that the fabric containing SSF is effective for shielding purpose and EMSE of fabric depending upon the orientation of t-ECCYs within fabric structure regarding the direction of the electrical field. With *SE*_T_ of less than 0.3 dB, t-ECCY-incorporated fabric is transparent to radiation in perpendicular polarization. A maximum *SE*_T_ of about 25 dB at parallel polarization was observed at 8.2 GHz in X-band. In addition, the *SE*_T_ seems to be independent to frequency from 8.2 to 10 GHz, after which it shows a decreasing trend with the increasing frequency. Furthermore, the *A*, *R* and *T* seem approximately constant as a function of the frequency for either of the polarizations, as shown in Figure 6c. In addition, fabric with SSF exhibited transmission (*T* > 95%) in the perpendicular polarization direction, whilst in parallel polarization effective shielding was observed (*T* ≈ 0).

To determine the contributions of reflection and absorption mechanisms to the overall EMI shielding performance of fabric containing SSF in parallel polarization of EM waves, the *SE*_A_ and *SE*_R_ values of the fabric were calculated and are shown in Figure 6d. It can be seen that *SE*_A_ is apparently higher than *SE*_R_ in frequency 8.2–10.6 GHz, which suggest that the contribution of the absorption is intrinsically dominant in the fabric. After that, an opposite trend was observed, demonstrating the reflection is the primary mechanism in frequency 10.6–12.4 GHz. In addition, *SE*_A_ decreases gradually while *SE*_R_ increases marginally over the entire X-band frequency range.

#### 4.2.2. EMSE of Fabric at Different Elongations

In practical applications, maintaining EMSE under mechanical conditions is very important in effectively protecting humans from EMI exposure. The EMSE of as-prepared weft-stretchable fabric containing SSF was therefore measured under three strain conditions (0%, 10% and 40%) according to its maximum measured stretch elasticity of fabric (≈ 42.6%) and the skin expansion at different body parts (i.e., 10%–50%) during human movement.

**Figure 7:**
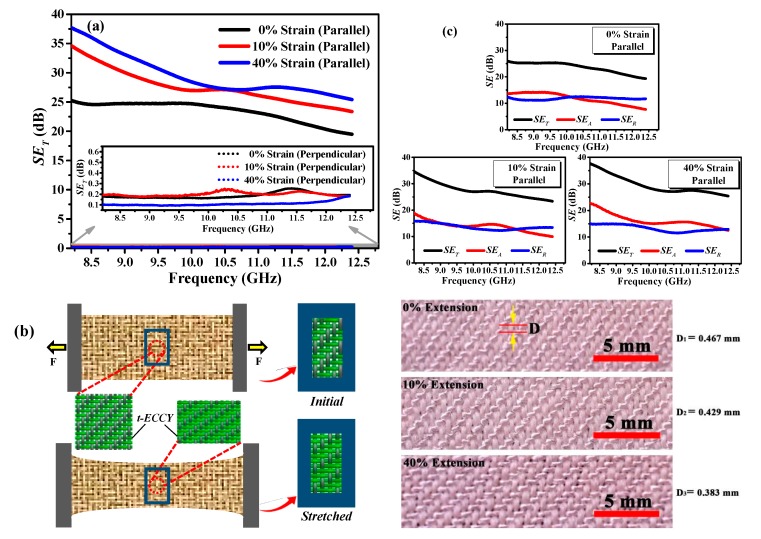
(**a**) Total EMSE results of stretchable fabric at different elongations, i.e., 0%, 10% and 40% extensions, at perpendicular and parallel polarizations of EM waves over X-band; (**b**) the corset phenomenon of a fabric during stretch and the distance of adjacent t-ECCYs at extensions of 0%, 10% and 40%; (**c**) the corresponding absorption and reflection contributions at parallel polarization at different extensions.

The *SE*_T_ of the weft-stretchable fabric containing SSF versus frequency at different elongations is presented in Figure 7a. As expected, with *SE*_T_ of less than 0.3 dB, the fabrics are obviously transparent to EM waves in perpendicular polarization irrespective of the applied strain considered, exhibiting almost no shielding ability. However, the highest EMSE values were recorded; 25.27, 34.62 and 37.68 dB at 8.2 GHz for parallel polarization. A frequency dependency of the *SE*_T_ shows a decreasing trend with increasing frequency. The EMSE value reaches above 30 dB at the 8.2–9 GHz and 8.2–9.6 GHz frequency ranges for parallel polarization at extension of 10% and 40%, respectively. Such values are regarded to be within the normal shielding range [49]. In addition, the value reaches above 20 dB over almost the entire X-band irrespective of the applied strains considered, indicating that the fabrics are highly suitable for general uses and perhaps many special/professional applications [50]. The EMSE capability of a fabric depends not only on the orientation of conductive yarns within the fabric structure but also the amount of the conductive yarns per unit area [47]. The SSF component inside t-ECCY within the fabric in initial state has a relatively loose and unstrained path, which increases the SSF path length [24]; hence, it has a higher electrical resistance, resulting in a lower *SE*_T_ [15]. The SSF length decreases with the continuous stretch of the fabric, e.g., 10% or 40% pre-strain, which, in turns, leads to lower electrical resistance and higher *SE*_T_. Furthermore, with an increasing extension, the distance between adjacent t-ECCYs, as observed with a USB digital microscope within the fabric along weft direction, gets smaller, slightly leading to a tighter SSF parallel array (see in Figure 7b). The reflection in parallel polarization is prominent; the blocking ability of EM wave enhances to some extent. Consequently, the EMSE of t-ECCY-incorporated fabric has significantly improved at elevated elongations at the parallel polarization.

The relative contributions of *SE*_A_ and *SE*_R_ of fabric in parallel polarization at 0%, 10% and 40% extensions to total *SE*_T_ are shown in Figure 7c. With the increasing applied strains, especially at 40% extension, the total *SE*_T_ is dominated by absorption because *SE*_A_ is apparently higher than *SE*_R_ almost over the entire X-band. Furthermore, with increasing strains, the *SE*_A_ increases remarkably with the biggest difference of above 9 dB at 8.2 GHz whilst the *SE*_R_ almost kept constant with the biggest difference of less than 3 dB at 8.2 GHz.

#### 4.2.3. EMSE of Fabric Following Cyclic Stretch

As seen in Table 1, the fabric has acceptable static elastic recovery value (80.83%) and stable dynamic work recovery value after fifteen stretch-release cycles.

**Figure 8:**
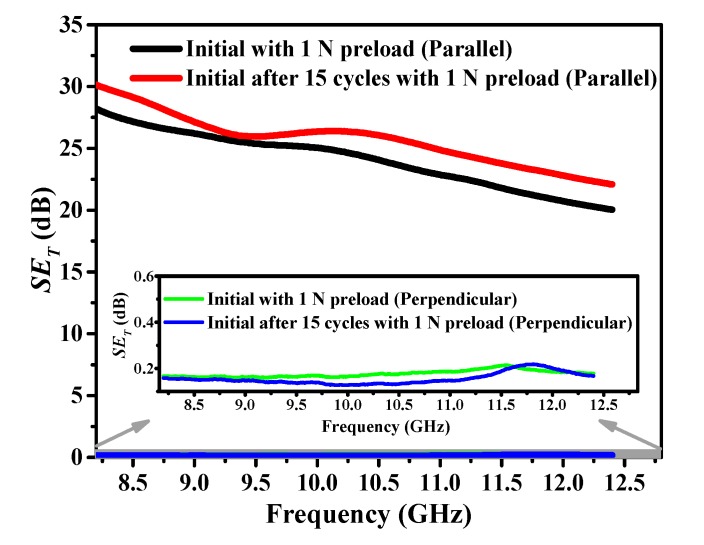
Total EMSE results of stretchable fabric with 1 N pretension in its initial state and after 15 cycles at perpendicular and parallel polarizations of EM waves in the X-band.

The subsequent *SE*_T_ of stretchable fabric containing SSF versus frequency following cyclic stretch is shown in Figure 8. The perpendicular polarization fabric containing t-ECCYs displayed no EMSE, because the perpendicular polarized EM waves could easily pass through the perpendicular slits in the fabric. The warp and weft directions represent two independent systems from each other concerning EMSE. The t-ECCY fabric displays effective EMSE capability, and the value increases with the fabric after 15 cycles. Fabric following cyclic stretch will cause certain creep elongation strain, similar to the analysis in Section 4.2.2., the shorter distance (SSF length) within a certain area of the fabric gets smaller, so it has a lower electrical resistance, resulting in a higher EMSE. The EMSE values under various conditions reach above 20 dB almost over the entire X-band range, indicating that these fabrics are highly suitable for most all general uses and many professional applications [50]. On the other hand, since the EMSE increases after 15 cycles, the difference of values between the initial and after 15 cyclic stretch states is not remarkable (less than 2 dB). In addition, the total *SE*_T_ shows a decreasing trend with increasing frequency.

#### 4.2.4. EMSE of Two-Layer Laminated Fabrics

If several layers of a t-ECCY containing woven fabric are stacked or laminated (layered) together, it is possible to shape the frequency response as desired. Herein, two fabrics were laminated in an appropriate order with varying lamination angles and polarization directions.

**Figure 9:**
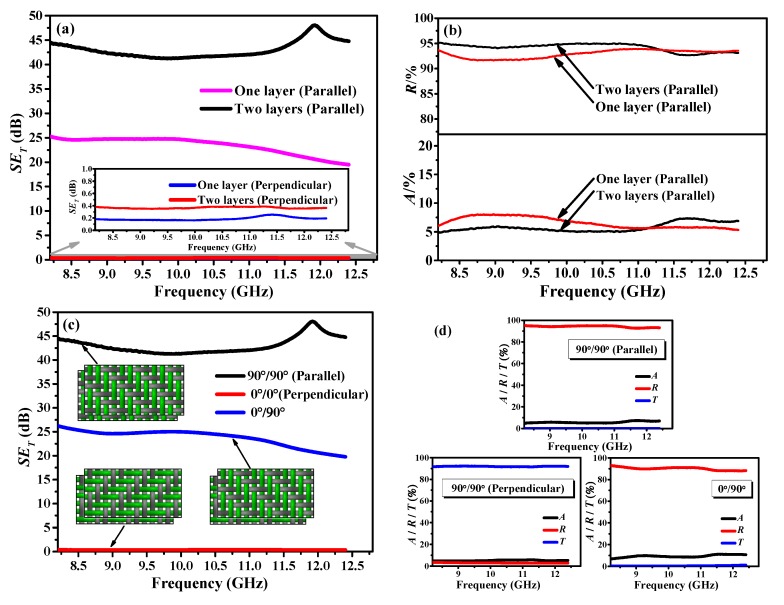
(**a**,**b**) Effect of lamination number on the total EMSE and the corresponding absorption and reflection coefficients of fabric with the same lamination angles, respectively; (**c**,**d**) Total EMSE and the respective absorption, reflection and transmission coefficients of two-layer fabric with varying lamination angles and polarization directions, respectively.

The effect of the number of layers on the *SE*_T_ values of the stretchable fabric containing SSF at both the perpendicular and parallel polarizations is presented in Figure 9a. Both the one- and two-layer fabrics do not have EMSE for perpendicular polarization, as expected. However, the fabrics have EMSE capability in the parallel polarization direction. The two-layer laminated fabric could remarkably improve EMSE ability compared to the one-layer fabric with its value almost twice higher. Furthermore, a remarkable EMSE peak of 48.06 dB was obtained at 11.92 GHz. The increased EMSE of the two layer-structured fabric was mainly based on the improved reflection (i.e., Value-*R* increased from 91.5%–93.6% to 93%–95.2%) rather than absorption (i.e., Value-*A* decreased from 5.3%–8.0% to 4.8%–7.2%), as presented in Figure 9b.

Figure 9c reveals the EMSE of two-layer laminated fabrics with different lamination angles (0°/0°, 0°/90°). By comparing Figure 9a,c, it can be concluded that the two-layer 0°/90° laminated fabric could not remarkably improve the EMSE compared to one-layer fabric, although a SSF metal grid has formed. In addition, the respective absorption, reflection and transmission coefficients (*A*, *R* and *T*) of two-layer fabrics with different lamination angles and polarization directions are presented in Figure 9d. The *A*, *R* and *T* seem approximately constant as a function of frequency under different testing conditions. For 90°/90° parallel polarization and 0°/90° orthogonal configuration, effective EM shielding was observed (*T* ≈ 0), whilst fabric exhibited transmission (*T* > 92%) at 0°/0° perpendicular polarization. In short, a two-layer 0°/90° laminated fabric was enough to satisfy people’s needs against EM waves in daily life, since the EMSE values were almost all above 20 dB in the entire X-band frequency range [49].

Considering the above analysis, since one-layered or two-layered fabrics had EMSE properties for electric field polarization in the same direction as the main direction of the conductive metal wires inside the fabric, it may not be suitable for the practical application because of the increase in fabric weight and mechanical interruptions as a result of the laminating. The results suggest the direct weaving of fabric made from 100% t-ECCYs (in the warp and weft) would be a useful way to ensure the desirable EMSE performance as well as superb stretchable elasticity.

## 5. Conclusions

Multifunctional composite yarns and such yarn-based products have proven to be essential in accomplishing recent stringent technical advancements. To integrate some intriguing features in a fabric, including electromagnetic shielding, flexibility, cyclic stretchability, durability and easy processing, herein, an ingenious design of a woven fabric containing tri-component elastic-conductive composite yarns (t-ECCYs) along its weft direction was prepared, and a comprehensive analysis of its EMSE behavior as a fabric under various conditions, e.g., single elevated elongations, cyclic stretch and lamination over the X-band range of frequencies, was carried out. Furthermore, the effect of perpendicular and parallel polarization of electromagnetic waves on EMSE was also studied.

The results reveal that the t-ECCY fabric has a denser (scrunched) surface compared with a pure cotton control, which facilitated weft stretchability up to a high elongation (>40%). Moreover, the fabric had excellent static and dynamic elastic recovery properties. It exhibited good 15-cyclic stability with 80.83% shape recovery retention and repeatable dynamic hysteretic behavior after approximately one training cycle.

The results show the EMSE of the t-ECCY fabric depended upon the orientation of t-ECCYs in the fabric structure to the direction of electric field. Differences between the EMSE of the t-ECCY fabric to the perpendicular and parallel polarization of EM waves was significant, exhibiting better results in parallel polarization. As expected, the control cotton fabric had poor EMSE irrespective of polarization, confirming that as an intrinsic insulating material, it was not suitable for EMI shielding. The EMSE of the t-ECCY fabric significantly improved at elevated elongations during single stretch primarily due to the lower electrical resistance occupied per unit area. Following cyclic stretch caused the EMSE value of the resulting fabric at pre-extension of 20% to increase slightly.

The double-layer fabric with 90°/90° lamination improved EMSE compared to its corresponding one-layer fabric, and the double-layer laminated fabric (0°/90°) had a value almost twice higher than the latter two. It is noteworthy to mention that, in general, EMSE decreases with increasing incident frequency, and hence, it can be predicted that when the frequency increases, the wavelength becomes shorter, and incident waves were able to penetrate through the pores of fabric.

The straightforward fabrication strategy and excellent EMSE performance witnessed in t-ECCY-based fabrics may find pervasive applications in satellite communications, radar navigation, and many other civil and military fields. Critically, there remains a myriad more combinations of yarns with different metal wires and fabric structures that need to be determined so that practical use of textile products can be achieved. Such work is fundamental to the next generation of highly flexible, stretchable and electromagnetic shielding composite fabrics.

## Figures and Tables

**Table 1:** Elastic recovery ratio (*ERR*) and plastic deformation ratio (*PDR*) values of fabric following cyclic tensile tests at an extension level of 20%.

Stretchable Fabric	1 Cycle		10 Cycles		15 Cycles	
	*ERR*	*PDR*	*ERR*	*PDR*	*ERR*	*PDR*
Specimen 1	95%	1%	82.5%	3.5%	77.5%	4.5%
Specimen 2	97.5%	0.5%	82.5%	3.5%	85%	3%
Specimen 3	90%	2%	85%	4%	80%	4%
Average Values	94.17%	1.17%	83.33%	3.67%	80.83%	3.83%
